# Multiple-statistical genome-wide association analysis and genomic prediction of fruit aroma and agronomic traits in peaches

**DOI:** 10.1093/hr/uhad117

**Published:** 2023-05-31

**Authors:** Xiongwei Li, Jiabo Wang, Mingshen Su, Minghao Zhang, Yang Hu, Jihong Du, Huijuan Zhou, Xiaofeng Yang, Xianan Zhang, Huijuan Jia, Zhongshan Gao, Zhengwen Ye

**Affiliations:** Peach Research Department of Forest & Fruit Tree Institute, Shanghai Academy of Agricultural Sciences, Shanghai 201403, China; Key Laboratory of Qinghai-Tibetan Plateau Animal Genetic Resource Reservation and Utilization (Southwest Minzu University, Ministry of Education), Chengdu, Sichuan 610041, China; Peach Research Department of Forest & Fruit Tree Institute, Shanghai Academy of Agricultural Sciences, Shanghai 201403, China; Peach Research Department of Forest & Fruit Tree Institute, Shanghai Academy of Agricultural Sciences, Shanghai 201403, China; Peach Research Department of Forest & Fruit Tree Institute, Shanghai Academy of Agricultural Sciences, Shanghai 201403, China; Peach Research Department of Forest & Fruit Tree Institute, Shanghai Academy of Agricultural Sciences, Shanghai 201403, China; Peach Research Department of Forest & Fruit Tree Institute, Shanghai Academy of Agricultural Sciences, Shanghai 201403, China; Peach Group of Shanghai Runzhuang Agricultural Science and Technology Institute, Shanghai 201415, China; Peach Research Department of Forest & Fruit Tree Institute, Shanghai Academy of Agricultural Sciences, Shanghai 201403, China; Department of Horticulture, Key Laboratory for Horticultural Plant Growth, Development and Quality Improvement of State Agriculture Ministry, Zhejiang Unihversity, Hangzhou 310058, China; Department of Horticulture, Key Laboratory for Horticultural Plant Growth, Development and Quality Improvement of State Agriculture Ministry, Zhejiang Unihversity, Hangzhou 310058, China; Peach Research Department of Forest & Fruit Tree Institute, Shanghai Academy of Agricultural Sciences, Shanghai 201403, China

## Abstract

‘Chinese Cling’ is an important founder in peach breeding history due to the pleasant flavor. Genome-wide association studies (GWAS) combined with genomic selection are promising tools in fruit tree breeding, as there is a considerable time lapse between crossing and release of a cultivar. In this study, 242 peaches from Shanghai germplasm were genotyped with 145 456 single-nucleotide polymorphisms (SNPs). The six agronomic traits of fruit flesh color, fruit shape, fruit hairiness, flower type, pollen sterility, and soluble solids content, along with 14 key volatile odor compounds (VOCs), were recorded for multiple-statistical GWAS. Except the reported candidate genes, six novel genes were identified as associated with these traits. Thirty-nine significant SNPs were associated with eight VOCs. The putative candidate genes were confirmed for VOCs by RNA-seq, including three genes in the biosynthesis pathway found to be associated with linalool, soluble solids content, and *cis*-3-hexenyl acetate. Multiple-trait genomic prediction enhanced the predictive ability for γ-decalactone to 0.7415 compared with the single-trait model value of 0.1017. One PTS1-SSR marker was designed to predict the linalool content, and the favorable genotype 187/187 was confirmed, mainly existing in the ‘Shanghai Shuimi’ landrace. Overall, our findings will be helpful in determining peach accessions with the ideal phenotype and show the potential of multiple-trait genomic prediction to improve accuracy for highly correlated genetic traits. The diagnostic marker will be valuable for the breeder to bridge the gap between quantitative trait loci and marker-assisted selection for developing strong-aroma cultivars.

## Introduction

Peach is one of the most economically important stone fruits and contains various flavor compounds. Due to the small genome size, it is recognized as a model fruit for other species in the Rosaceae family. The origin of peach and long domestication history of its cultivation in China has resulted in a high level of genetic diversity [1]. Global peach production was estimated at 21 million tons in 2021, 78% of which was from China (Food and Agriculture Organization of the United Nations, 2021).

Fruit flavor is essential to consumer preference and is becoming a hotspot in plant breeding programs [2]. It is determined by various compounds, including volatiles, sugars, acids, and nutritional compounds [3]. These compounds have always been identified as quantitative traits controlled by many small-effect genes or quantitative trait loci (QTLs) [4]. Fruit aroma is one of the quality factors influencing consumer appreciation [5]. While more than 100 volatile odor compounds (VOCs) have been identified in peach, only around 20 compounds have odor activity values higher than 1 and are putatively considered as key in peach aroma. Among these volatiles, lactones, in particular γ-decalactone, appear to be the main contributors to peach notes. Esters such as (*Z*)-3-hexenyl acetate and (*E*)-2-hexenyl acetate mainly contribute ‘fruity’ notes, while terpenoid compounds like linalool and β-ionone provide ‘floral’ notes. (*Z*)-3-hexenal and (*E*)-2-hexenal have been described as ‘green or grass’ notes [6–8]. As in other fruit species, although great success has been achieved in selective breeding for larger fruits, high yield, increased disease resistance, and better postharvest performance for long-term storage or shipping, this might indirectly decrease fruit aroma [5, 9, 10]. Some volatiles have even been lost during domestication and selection [2, 10–13]. For example, breeders introduced wild species as parental material for resistant cultivar selection, and these species always have less fruit flavor. On the other hand, firmer fruit texture was always linked to less ethylene production, which is a key regulator for volatile compound formation [14]. It is necessary to identify the associated genomic regions for major VOCs to develop valuable genomic tools for marker-assisted selection (MAS) and genomic selection (GS), and to enhance prediction accuracy and efficiency in modern peach breeding programs.

With the decrease in genotyping costs and improved analytical approaches, approaches based on next-generation sequencing technologies, such as genome-wide association study (GWAS), have proved to be efficient methods to understand the genetic basis of quantitative agronomic traits. Association mapping identifies significant signals between marker phenotypes in the population. A large number of GWAS studies have been reported in several fruit tree species [10, 11, 15–17]. For peach, the genome was released by the International Peach Genome Initiative in 2013, and then improved in 2017 [18, 19]. Based on this, GWAS has been used for many traits, including fruit shape, fruit color, fruit hairiness, fruit weight [15], sugar, acid and phenolic compounds [16], and disease [17, 20]. However, it has rarely been used for fruit volatiles, controlled by multiple genes of small effect [21]. MAS in peach is still restricted to a limited number of traits associated with single genes or large-effect QTLs.

GS (also referred to as genomic prediction) was first proposed by Bernardo and Meuwissen *et al*. [22, 23]. It is becoming an established methodology in both animal and plant breeding programs to predict the genetic value of untested lines, based on genome-wide DNA variation together with phenotypic information on an observed population [4]. Genomic estimated breeding value (GEBV) can be used to evaluate the genetic potency of lines in the early stages of growth and development, which can be determined by three major methods: Bayesian, best linear unbiased prediction (BLUP), and deep learning methods. The prediction accuracies of these three approaches are always affected by the size of the training population, relationships between individuals, marker density, and genetic architectures [4]. Most genomic selection studies in plants have been on annual crops such as spinach [24], common bean [25], soybean [26, 27], and wheat [28], while few have been published in fruit species [21, 29–32]. The first GS study in apple reported that the genomic accuracies for six fruit quality traits varied from 0.70 to 0.90 [29]. With a multi-environment apple reference population in European research groups, 59 stable genome-association regions of 30 quantitative traits have been identified by GWAS. The average genomic predictive abilities ranged from 0.18 to 0.88. In peach, the IPSC 9 K single-nucleotide polymorphism (SNP) array v1 was used to genotype 1147 European peach individuals to predict the accuracy for fruit weight, sugar content, and titratable acidity. The genomic predictive abilities were as high as 0.84 for fruit weight and 0.83 for titratable acidity, which suggested GS could be a promising approach in peach breeding programs [31]. GS models in volatile compounds have not yet been reported in peach due to the difficulty of phenotype assessment. All these studies strongly suggest that GS could be a credible and effective solution to obtain GEBV for choosing elite parents or seedlings as potential commercial cultivars at a very early stage [29].

Here, we integrated an omics approach combining gas chromatography–mass spectrometry (GC–MS), multiple-statistical GWAS, genomic prediction (GP) and MAS to uncover the genetic architecture of six agronomic traits and 14 fruit VOCs with a diverse germplasm. The significant association signals of eight major VOCs were first used in GP and transformed into DNA markers to investigate the allelic variation. Finally, the desirable and undesirable genotypes for floral-note linalool were selected for being useful in target selection of superior flavor cultivars by combining MAS and the comprehensively evaluated phenotype database.

## Results

### Phenotype variation and correlation among different traits

**Figure 1:**
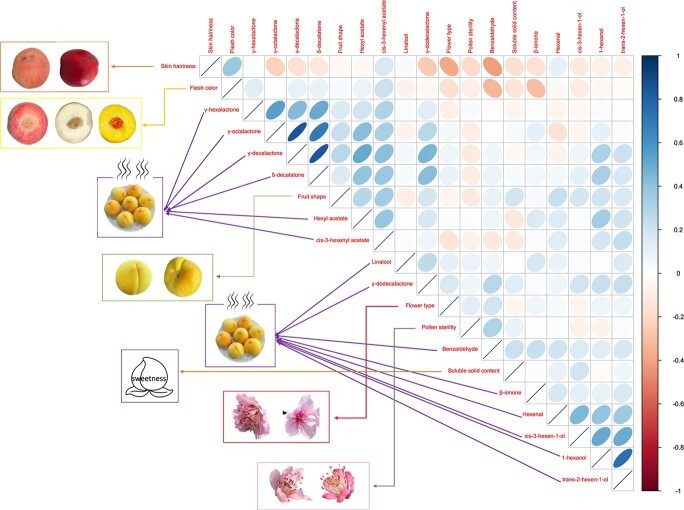
. Heat map showing correlations of all phenotypes across peach accessions.

Here we constructed a comprehensive phenotypic database for six agronomic traits in 242 accessions curated in Shanghai, and 14 aroma-related VOCs in 184 accessions (Supplementary Data Table S1, Fig. 1). The agronomic traits included fruit flesh color, fruit shape, fruit hairiness, flower type and pollen sterility, and soluble solids content related to fruit sweetness. The 14 characteristic VOCs included two esters as fruity note; five lactones as peach note; hexanal and three C6 alcohols as grass note; linalool and β-ionone as floral note; and benzaldehyde as almond note. All qualitative traits were displayed as binomial distributions, and most quantitative traits had a normal distribution (Supplementary Data Fig. S1). The VOC content varied greatly between different traits and accessions. The accessions containing the highest amount of chemical were identified for each trait. For example, the content of *cis*-3-hexenyl acetate ranged from 0.020 to 440.37 ng/g with a mean value of 55.07 ng/g, with the highest content in cv. ‘Okubo’, which is an elite backbone parent in peach breeding programs. The highest level of linalool was found in the well-known flat nectarine ‘Jin Xia Zao You Pan’ and the lowest in the stony-hard cv. ‘Gui Fei’. The total content of lactone ranged from 1.42 to 1674.85 μg g^−1^ fresh weight (FW), and the highest level was observed in ‘Qiu Yue’. Stony-hard cultivars accumulated much less lactone compared with melting accessions, including ‘Gui Fei’, ‘Dai Fei’, ‘Feng Bai’, ‘Cui Bao’, ‘Yan Bao’, ‘Zhong You 20’, and ‘Yan Bao’.

The volatile content was further compared between different peach groups separated according to agronomic traits (fruit shape, flesh color, skin hairiness, fruit texture) and domestication history (Supplementary Data Table S1). Flat peach accumulated much higher content of volatiles than round peach. Yellow peach accumulated higher lactones and *cis*-3-hexenyl acetate than white and red flesh peaches. Red flesh peach accumulated much higher β-ionone and C6 alcohols and aldehyde. Peach accumulated higher lactones and less linalool compared with nectarine. Apart from linalool, hexenal, and *cis*-3-hexenol, all the other volatiles were higher in melting peach than in stony-hard peach. All volatiles were higher in traditional landraces than in improved cultivars.

There were significant (*P* < .01) correlations between different traits (Supplementary Data Fig. S1). Pearson’s correlation coefficients between pairwise traits ranged from −0.38 to 0.85. As expected, there was always a strong correlation between the biosynthetically related chemicals. For example, the correlation between δ-decalactone and γ-decalactone was as high as 0.85, and the correlation between 1-hexanol and trans-2-hexen-1-ol was 0.75. All lactones evaluated in this study positively correlated with each other. It is interesting to note that the qualitative trait fruit shape showed a relatively higher and positive correlation with the contents of *cis*-3-hexenyl acetate, hexenyl acetate, and γ-decalactone, with correlation values of 0.35, 0.28, and 0.27 respectively. Fruit color showed relatively higher correlation with the content of β-ionone and benzaldehyde. Due to the low content of lactones in the stony-hard cultivar, we additionally analyzed the correlations between each lactone and fruit texture (melting/stony-hard). The result showed a weak and negative correlation, with the coefficient ranging from −0.138 (γ-decalactone) to −0.26 (γ-dodecalactone).

### Genome-wide association analysis of six agronomic traits and candidate gene identification

**Figure 2:**
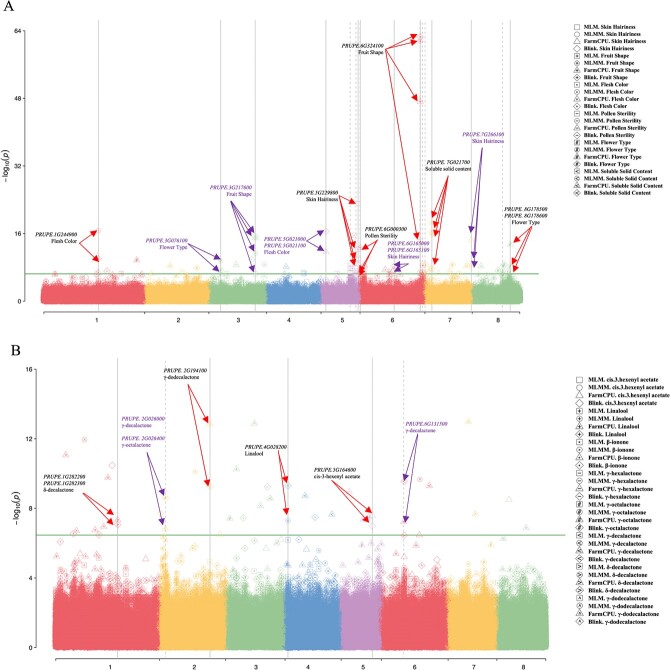
. Manhattan plot of GWAS for 14 traits. On the right, the shapes of the symbols indicate the statistical model used for the association analysis, and the labels within the graphic symbol (such as ‘*’) indicate the different traits. (A) Manhattan plot of GWAS for six agronomic traits based on four statistical association models. The green horizontal line represents the significance threshold (−log_10_*P* = 7). A red arrow indicates that the associated locus is close to the published loci, and a purple arrow indicates that the associated SNP is a novel locus. (B) Manhattan plot of GWAS for eight VOCs based on four statistical association models. The *x*-axis represents the eight peach chromosomes and the *y*-axis represents −log_10_ (P). A red arrow indicates that the locus is associated with only one trait and identified by multiple GWAS models. A purple arrow indicates that the locus is a hotspot that is associated with several traits.

Using four statistical models, GWAS was carried out for all investigated traits. A total of 60 significant SNPs were found to be associated with six agronomic traits, including 14 SNPs for soluble solid contents and 46 SNPs for five quanlitative traits. Ten SNPs were commonly detected by at least two models (Supplementary Data Table S2, Fig. 2A). The number of candidate genes for each trait ranged from 137 (pollen sterility) to 397 (skin hairiness) (Supplementary Data Table S3).

Eleven SNPs were significantly associated with fruit flesh color. The SNP rs38148 on chr 1 was identified by the two statistical methods, and is located within *PRUPE.1G244900*, which encodes a leucine-rich repeat. Expression of this gene was significantly higher in the ripening fruit of yellow-flesh cv. ‘Jin Chun’ than in the white-flesh cv. ‘Hikawa Hakuho’. The most important flesh color regulating gene, *carotenoid cleavage dioxygenase 4* (*CCD4*, *PRUPE.1G255500*), was located at ~800 kb downstream of SNP rs38148. A novel SNP rs214574 on chr 5 was mapped by BLINK (Bayesian information and linkage disequilibrium iteratively nested keyway) and FarmCPU (fixed and random model circulating probability unification), and the nearest flanking candidate genes were *PRUPE.5G021100*, encoding *S*-adenosyl-l-methionine-dependent methyltransferase*.* RNA-seq gave a different expression pattern of this gene in yellow-flesh peach cv. ‘Jin Chun’ and white-flesh peach cv. ‘Hikawa Hakuho’ during the developmental and ripening stages.

Six SNPs were significantly associated with fruit shape, and two of them were identified by four statistical models. They were SNP rs287040 and SNP rs157802. The former was located within a *lipoxygenase* gene of *PRUPE.6G324100* on chr 6: 28554097–28558317 bp. Above 98% flat accessions with genotype CT could be distinguished by the SNP in Pp06: 29760757 bp. The SNP rs157802, located within *PRUPE.3G217600* on chr 3, was a novel locus.

As for skin hairiness, the SNP rs239188 at chr 5: 17571575 bp was detected by four models with the minor allele frequency (MAF) value of 0.269. Around 160 kb upstream of this SNP, a candidate gene *PRUPE.5G196100* was found on chr 5: 15892316–15894063 bp. This gene is an *R2R3-MYB* member that has been reported to regulate trichome development and cuticular wax biosynthesis in *Prunus persica*. Two novel associated SNPs were also detected by multiple models, rs266998 at chr 6: 16212705 bp and rs328418 at chr 7: 21947093 bp. On chr 7, three candidate genes, *PRUPE.7G075300* (*MYB-like DNA-binding domain*), *PRUPE.7G075500* (*Cotton fiber expressed protein*), and *PRUPE.7G265400* (*Cellulose synthase*), were identified within 40 kb around the SNPs. These were differentially expressed genes in nectarine and peach, according to a previous report [33]. We also detected three *HD-ZIP* genes flanking the SNP rs328418: *PRUPE.7G082300*, *PRUPE.7G264500*, and *PRUPE.7G079300*.

Concerning two flower-related traits, nine SNPs were significantly associated with showy/non-showy flower type, of which five were located on chr 8: 12261040–17987622 bp, and near to the reported regions. Two novel SNPs (rs136646 and rs356147) were mapped at chr 3: 5594494 bp and chr 8: 17987622 bp by more than two statistical models. The former SNP linked with three *ABC-2 transporter* genes, one of which has been validated as controlling flower cuticle secretion and the pattern of the petal epidermis in *Arabidopsis* [34]. Eight SNPs were identified as associated with pollen sterility. Three were located on chr 6, and the SNP rs241019 within *PRUPE.6G000300* was identified by three models with the MAF value of 0.347.

Fourteen SNPs were associated with soluble solid contents, and nine of them were located on chr 7. The SNP rs297494 on chr 7: 3308817 bp was detected by all statistical models. Candidate gene searching showed that it was located within *PRUPE.7G021700*, with extremely low expression during peach developmental stages. In addition to this, in the upstream region of this SNP we identified three differentially expressed genes encoding α-l-arabinofuranosidase during fruit developmental stages.

Using a group of significant markers mainly selected from the hotspot region for each trait, we estimated the percentage of phenotype explained by different genotype combinations (Supplementary Data Fig. S2). The percentage of phenotype under different genotype combinations was 94% for pollen sterility, 98% for fruit shape, and 98% for skin hairiness. However, the percentage was relatively low for flower type and flesh color. As a quantitative flavor-related trait, the phenotype distribution of soluble solids content was compared with different genotypes of SNP rs297494. The mean value was significantly higher under the genotype GA than that under GG (Supplementary Data Fig. S3).

### Genome-wide association analysis of eight volatile odor compounds and candidate gene identification

Association mapping of 184 accessions was based on the phenotype of eight aroma volatiles (Fig. 2B, Supplementary Data Table S4). A Manhattan plot showed that 39 SNPs above the threshold were significantly associated with eight VOCs. The highest number of associated signals was on chr 1. The number of candidate genes predicted for each trait was from 24 (γ-octalactone) to 172 (γ-decalactone) (Supplementary Data Table S5). The Pearson correlation (*P* < .05) between the transcript level of candidate genes and the change of VOC content of the two cultivars was calculated to find the most correlated genes (Supplementary Data Fig. S4, Supplementary Data Table S5). The genomic region within a 300-kb window associated with more than one trait was defined as a hotspot region. A total of 136 candidate genes were identified in two hotspot regions (Supplementary Data Table S6).

**Figure 3:**
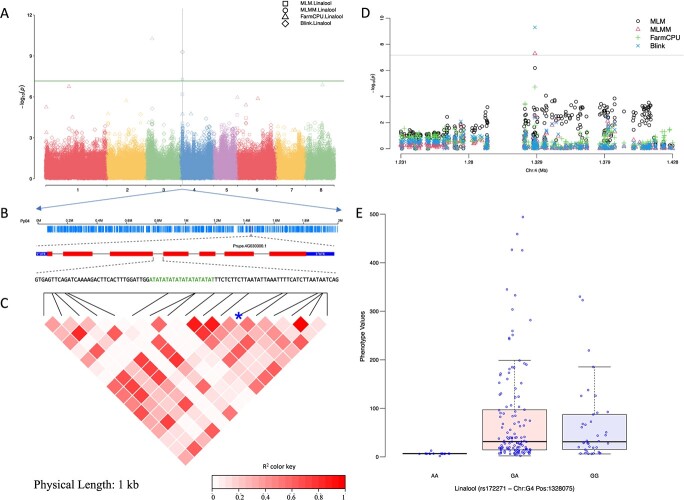
Significant locus on chromosome 4 related to linalool. (A) Manhattan plot shows the significant SNP rs172271 at the genome-wide level. The vertical dotted-line indicates the position of the significant locus. (B) Gene structure of the annotated candidate genes. (C) The LD block shows the pairwise correlation between the SNPs within the window of 1 kb along the significant genomic region. The blue asterisk indicates the position of the significant SNP rs172271. (D) Manhattan plot shows the significant SNP rs172271 at the chromosome level. (E) The box plot shows the comparison of the phenotypic distribution under different genotypes.

As for the two ‘floral-note’ compounds, three SNPs, on chr 1, 3, and 4, were identified as associated with β-ionone. Forty candidate genes were identified within the flanking region. Six SNPs on chr 1, 3, 4, and 8 were significantly associated with linalool. The SNP rs172271 at the genome position of chr 4: 1328075 bp was detected by three models. Downstream of this locus, three *terpene synthase* genes (*PRUPE.4G029900*, *PRUPE.4G030300*, and *PRUPE.4G030400*) were found to be located from position 1390452 to 1422386 bp. RNA-seq analysis showed there was almost no expression of *PRUPE.4G029900* in peach fruit. The accumulation of linalool in two peach cultivars correlated positively with the expression of *PRUPE.4G030300* and negatively with the expression of *PRUPE.4G030400* [35] (Fig. 3, Supplementary Data Fig. S5)*.*

Considering the VOCs derived from the β-oxidation pathway, five SNPs were associated with the ‘fruity-note’ compound *cis*-3-hexenyl acetate, and SNP rs232930 on chr 5 was identified by both BLINK and FarmCPU. Another important SNP, rs76610, was located within *PRUPE.2G005300*, encoding lipoxygenase, with a significantly reduced pattern of expression during fruit developmental and ripening stages in two peach cultivars. Sequencing alignment by IGV (Integrative Genomics Viewer) identified two polymorphic SSRs in this gene (Supplementary Data Fig. S6). There were 27 SNPs associated with the five highly correlated ‘peach-flavor’ lactones. One SNP rs42799 between *PRUPE.1G282200* and *PRUPE.1G282300* on chr 1 was found to be associated with δ-decalactone by BLINK and FarmCPU. Another significant SNP, rs112565, located within the *PRUPE.2G194100* gene, was found to be associated with γ-dodecalactone by both BLINK and FarmCPU. The function annotation of *PRUPE.2G194100* was *cytochrome b5-like Heme/Steroid binding domain*. Twelve SNPs were associated with the major peach flavor contributor γ-decalactone, and three of them were found in two hotspot regions: two SNPs were located on chr 6: 10325269–10506244 bp associated with γ-decalactone, and the third one, located on chr 2: 2663753–2721214 bp, was associated with γ-decalactone and γ-octalactone.

**Figure 4:**
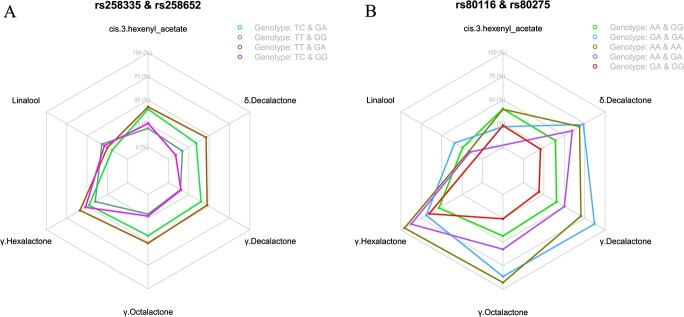
Radar graphic showing the percentage of the mean value of each phenotype of the whole population under different genotype combinations in two hotspots. Different colors indicate different genotype combinations.

For these correlated traits, the most favorable genotype combination of two hotspots was detected by investigating the phenotype distribution under different genotype combinations. The explained ratio of each phenotype is indicated by the percentage of the mean quantitative value in the whole population (Fig. 4). All lactone contents were highest under the genotype combination TT + GA in the hotspot region rs258335 + rs258652 (Fig. 4A). The average content of the most important peach aroma contributor, γ-decalactone, was highest when both genotypes were heterozygous for GA in the hotspot rs80116 + rs80275 (Fig. 4B).

### Accuracy of genomic prediction

**Figure 5:**
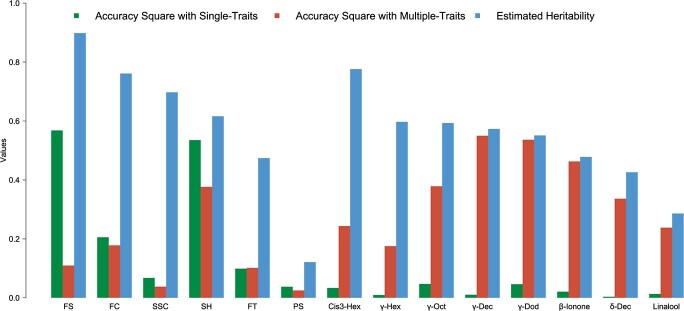
Explanation value of estimated heritability by squared accuracy value. Comparison of accuracy and estimated heritability for 14 traits. The squared value of accuracy indicates the proportion of the GP model explaining heritability. On the *x*-axis: FS, fruit shape; FC, flesh color; SSC, soluble solids content; SK, skin hairiness; FT, flower type; PS, pollen sterility. This is followed by *cis*-3-hexenyl acetate, γ-hexalactone, γ-octalactone, γ-decalactone, γ-dodecalactone, β-ionone, δ-decalactone, and linalool.

Both single-trait and multiple-trait GP models were used for 14 target traits as an aid for breeders to select potential elite accessions by using the significant markers obtained from GWAS. By using these markers for six lowly correlated qualitative traits, the genomic prediction accuracy ranged from 0.19 to 0.75, with the accuracy of the single-trait model higher than that of the multiple-trait model (Table 1). Considering that the biosynthesis of β-ionone is catalyzed by *CCD4* and highly correlated with fruit flesh color, we combined the genotype matrix of significant markers of the flesh color trait and β-ionone in the reference population to predict the GEBVs of individuals in the inference population. The prediction accuracy dramatically improved from 0.14 with the single-trait model to 0.68 with the multiple-trait model (Table 1). With the high Pearson correlation efficiency of fruit flavor-related traits (*cis*-3-hexenyl acetate, γ-hexalactone, γ-octalactone, γ-decalactone, δ-decalactone, and γ-dodecalactone) (Supplementary Data Fig. S1), the genotype matrix of the significant markers of these six traits were included as covariates in the GP model, enhancing the accuracy of prediction almost three times with the multiple-trait GP model. The narrow-sense heritability for nine flavor traits ranged from 0.426 for δ-decalactone to 0.776 for *cis*-3-hexenyl acetate. The estimated heritability by the GP approach was much higher for quantitative traits, especially for γ-decalactone, γ-dodecalactone, δ-decalactone, and β-ionone (Fig. 5).

**Table 1:** Genomic accuracy between observed and predicted phenotypes. All GPs were based on the significant markers from GWAS in the cross-validation. Accuracy refers to the correlation between all observed and predicted phenotypes.

Traits	Accuracy with multiple traits	Accuracy with single trait	*h* ^2^
Skin hairiness	0.6136	0.7315	0.616
Fruit shape	0.3309	0.7537	0.898
Flesh color	0.4217	0.4531	0.761
Pollen sterility	0.1588	0.1937	0.121
Flower type	0.3186	0.3142	0.474
Soluble solids content	0.1941	0.2598	0.697
β-Ionone	0.6801	0.1439	0.478
*cis*-3-Hexenyl acetate	0.4936	0.1824	0.776
γ-Hexalactone	0.4189	0.0968	0.597
γ-Octalactone	0.615	0.2168	0.593
γ-Decalactone	0.7415	0.1017	0.573
δ-Decalatone	0.5798	0.0575	0.426
γ-Dodecalactone	0.7323	0.2152	0.551

### Development, validation, and application of diagnostic DNA markers

**Figure 6:**
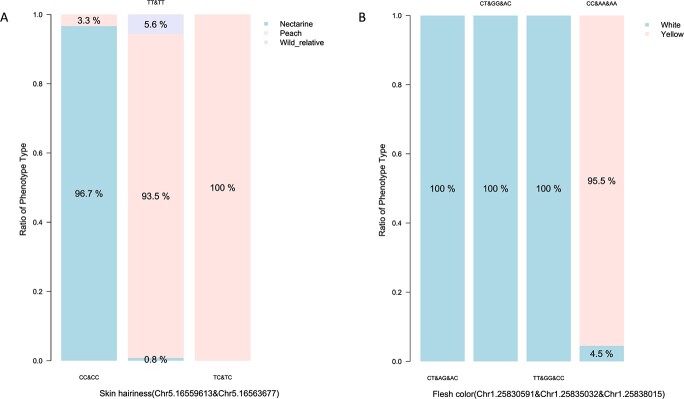
. Accuracy value between genotype and phenotype in testing populations with newly designed KASP markers based on associated loci for skin hairiness (A) and flesh color (B).

Based on the significant SNPs from the multiple GWAS model and IGV visualization, eight KASP (kompetitive allele-specific PCR) markers from the significant region were developed and validated to predict skin hairiness and flesh color in 242 randomly selected accessions, including 87 cultivars, 148 seedlings and 7 wild relatives. Five KASPs were selected to determine the fuzzy skin of peach fruit (Supplementary Data Table S7). The results showed that the genotype combination of peaches was TT/TT or TC/TC and for nectarines this was CC/CC on the two KASPs on chr 5. The accuracy between genotype and phenotype was 96.7% for skin hairless (nectarine), and 99.1% for fuzzy skin (peach and wild relatives) (Fig. 6A). The second group of KASPs was located on chr 7. They were closely linked and formed three genotype combinations. It is interesting to note that all three genotype combinations were observed in the peach group, but only two in the nectarine group. As for the three KASPs for flesh color, a total of eight genotype combinations were identified, four of which were unique genotype combinations. All 128 yellow accessions had a genotype combination of CC/AA/AA. However, 6 out of 107 white accessions were unexpectedly found to be CC/AA/AA. The accuracy between genotype and phenotype was 95.5% without considering the seven wild relatives and four unique GCs (Fig. 6B, Supplementary Data Table S8).

For the linalool-related marker validation, the SSR marker PTS1-SSR on chr 4: 1414653–1414673 bp was developed in the third intron of *PRUPE.030300*. A total of seven alleles (177, 179, 181, 183, 185, and 187 bp) were amplified and combined into 13 genotypes in 127 accessions. The most frequent was the 177 allele, with a frequency of 42% (111/254), followed by that of 187 bp, with an allele frequency of 26% (67/254). Two alleles (179 and 181) were rare alleles, only detected in ‘Hei Tao’, ‘Gan Xuan 2’ and ‘Long1-2-4’. Among all the genotypes, 177/177 was the most enriched genotype, followed by 177/183. One-way ANOVA pairwise-genotype comparisons showed that the average linalool content of 187/187 was remarkably high compared with other genotypes. This genotype mainly existed in ‘Shanghai Shuimi’ landraces: ‘Sa Hua Hong Pan Tao’, ‘Bai Hua’, ‘Chen Pu Pan Tao’, ‘Niu Ti Xing Pan Tao’, and ‘Sheng Ze Pan Tao’ (Supplementary Data Fig. S7).

## Discussion

One of the important objectives for peach geneticists and breeders is to enhance fruit quality, especially flavor, to satisfy the consumer’s eating experience and continued consumption. In this study, we integrated omics approaches to elucidate the genetic determination of key breeding traits, particularly aroma, with a group of peaches mostly derived from ‘Chinese Cling’, which has a strong aroma. The constructed phenotype database, including key VOC profiling as well as simple agronomic traits, provided comprehensive information to select superior cultivars as ideal breeding material or new selections. Considering the comparison of VOCs in different peach groups, some of our results are in agreement with a previous study [36]. For example, nectarines have more linalool and less lactone than peach, melting peach has higher lactones than the stony-hard type, and white peach has more β-ionone than yellow-flesh peach [37]. In the present study, almost all VOCs were higher in flat than in round peach, except benzaldehyde and β-ionone, suggesting that flat peach may be strong aroma material for breeding. An important finding is that all investigated VOCs were higher in traditional landraces than in improved cultivars, confirming that modern breeding strategies reduce peach fruit aroma to some degree [11], despite the major achievements with other traits. This indicates that certain landraces can be used to introduce new/elite aroma-related genes and to select strong or specific aroma-type peaches. Besides the association loci being consistent with previous reports, novel genes were identified with the multiple GWAS model, especially hotspot genomic regions for characteristic VOCs. A multiple-trait GP model was first applied in accuracy prediction of peach VOCs, which showed promise in biosynthetically related lactones. Based on the availability of genotypes and phenotypes in the test population, two types of diagnostic markers were developed and validated for skin hairiness, fruit flesh color, and linalool content, to evaluate their potential in MAS. The greatest advantages in using KASP markers are convenience, economy, high throughput, and efficiency. The combination of these associated markers can also be transformed to an applicable targeted genotyping array to test different traits in hundreds of thousands of new seedlings in the peach breeding process [37].

One of the targets of this study was to identify novel association signals that are related to six agronomic traits with traditional germplasm in Shanghai. As expected, most major loci we found are highly consistent with previous studies [15, 38], but we did find six novel genes for fruit flesh color (chr 5), fruit shape (chr 3), skin hairiness (chr 6, 7), flower type (chr 3), and soluble solids content (chr 7), with more than two statistical GWAS models. Taking skin hairiness as an example, peach pubescence is controlled by the single G locus [39], within which a functional fuzzy gene, *PpMYB25* (*PRUPE.5G169100*), has been reported to induce fruit trichome development and cuticular wax accumulation by activating the transcription of its downstream homolog, *PpMYB26* (*PRUPE.5G240000*) [33]. Here we not only accurately mapped these two genes, but also identified two novel loci (including six candidate genes) on chr 7. The differential expression of the three genes *PRUPE.7G075300* (*MYB-like DNA-binding domain*), *PRUPE.7G075500* (*Cotton fiber expressed protein*), and *PRUPE.7G265400* (*Cellulose synthase*) between nectarine and peach has been reported in Yang *et al*.’s research [33], but their gene function has not been validated. Other flanking genes newly identified were three *HD-ZIP* transcription factors, which play important roles in controlling fiber initiation in cotton [40] and the formation of glandular secretory trichomes in *Arabidopsis* [41], melon [42], and tomato [43]. Sequencing alignment by IGV showed that three SNPs in *PRUPE.7G264500* were highly linked with each other and formed three genotype combinations (Supplementary Data Table S9). However, only two genotype combinations were observed in nectarines in both the training and the testing population. All this evidence suggests that *HD-ZIP* at chr 7: 21.8 Mb might be a new factor regulating peach trichome formation.

The main aim of our study was the genetic dissection of fruit aroma based on our germplasm, since it is one of the most direct traits that influence consumer preference. QTL mapping of VOCs has been reported by several research groups, based on biparental [44, 45] and natural populations [11]. Eduardo *et al*. found 73 QTLs for 23 VOCs, with three major loci related to nonanal, linalool, and *p*-menth-1-en-9-al [44]. Sánchez *et al*. identified three loci on linkage groups 4, 5, and 6 that control monoterpene, lactone, and ester contents [45]. Cao *et al*. identified seven hotspots with more than four QTLs within a 1-Mb window, and found that the top end of chromosome 6 was related to benzaldehyde and δ-decalactone [11, 44]. Consistent with the aforementioned research [6, 11, 44], we mapped the same associated loci for the sweet and floral-note compound linalool. Fortunately, the SSR marker with allelic variation was detected and the most favorable genotype was confirmed as 187/187 bp. It is interesting to note that this genotype was mostly found in traditional landraces from Shanghai. This might be a good explanation why ‘Chinese Cling’ (also called ‘Shanghai Shuimi’) peaches have been the most favorable founder for groups of elite cultivars (such as ‘Jinxiu’, ‘Elberta’, ‘Redheaven’, ‘Okubo’, and ‘Hakuho’) in China, Japan, the USA, and Europe [46]. To a certain extent, the successful validation of this diagnostic marker further bridges the gap between identification of loci of volatiles and MAS, so it could be useful in high-floral-note cultivar innovation in peach breeding programs. Regarding the most important peach-like contributor, γ-decalactone, we did not identify the same or similar genome region as in the previous study. The predicted genes, especially highly correlated genes and the expression pattern, will provide more insight to explain lactone variation in different genotypes. Here a putative *LOX* gene, *PRUPE.2G005300*, was identified, and the gene expression was negatively related to *cis*-3-hexenyl acetate. The role of the *LOX* gene in ester and lactone formation has been reported in several Rosaceae species [47–51]. Validating the gene function by overexpression assays in tobacco (*Nicotiana benthamiana*) leaves via *Agrobacterium*-mediated transient transformation showed decreased *E*-2-hexanal compared with the empty vector control. Since *cis*-3-hexenyl acetate could not be detected in tobacco, we plan to repeat this experiment in tomato or strawberry in the future. The two polymorphic SSRs within this gene will be designed as new functional diagnostic markers to validate the allele effect in predicting phenotype accuracy.

Developing a new peach cultivar is a time-consuming process because of the long juvenile stage, large plant size, and the multi-year recording of phenotypic performance [52]. Nowadays, GS has been proved to be a promising tool for breeders by using whole-genome markers to predict the GEBV of individuals. Research in annual crops has shown that multiple-trait genomic selection is more capable than the single-trait genomic model of improving prediction, especially when phenotypes are not available for all individuals and traits [42, 53, 54]. In this study, prediction accuracy was fairly different among different traits. The application of multiple-trait genomic selection effectively improved prediction accuracy for these biosynthetically related VOCs by integrating these common loci, but the effect is the opposite for the non-correlated qualitative traits. This is a good indication that multiple-genomic selection could be an appropriate model for the prediction of genetically correlated complex traits in peach. It will be possible to improve prediction accuracy for the trait of interest in the inference population by using values of a tested phenotype as the covariate in the predicted model [55]. The accuracies of eight VOCs were from 0.4936 (*cis*-3-hexenyl acetate) to 0.7415 (γ-decalactone) by applying the multiple-trait GS model. The prediction value seems to be relatively lower than that from Biscarini *et al*.’s study on fruit weight (0.84) and titratable acidity (0.83) in peach [31]. However, according to the heritability equation and genetics theory, the squared value of predicted accuracy cannot exceed the estimated heritability. In this study, the estimated heritability for each volatile ranged from 0.426 to 0.776, which means the highest value of the predicted accuracy should be lower than 0.776. We estimated the range of VOCs’ heritability as similar to that of 23 volatiles in apple (from 0.15 to 0.85) [56]. Another reason is that we only used markers above the Bonferroni threshold from GWAS results. In most cases the use of significant markers could predict much higher phenotypic variation explained(PVE), but still not explain all heritability [57].

### Conclusions

Multiple GWAS models combined with transcriptomic profiling allowed us to accurately find novel association loci for complex aroma volatiles as well as traditional agronomic traits. The high genomic prediction accuracy based on GWAS-identified markers provides encouraging evidence to apply a multiple-trait genomic prediction model for genetically correlated traits. The high accuracy of diagnostic markers, particularly for linalool content, further bridges the gap between QTL detection and application of MAS. The newly designed KASP markers will be more suitable for high-throughput MAS implementation. Overall, these findings provide insight for candidate gene function validation, GS, and MAS.

## Materials and methods

### Plant materials

A total of 242 peach accessions were selected from the germplasm repository in Zhanghang experimental field of Shanghai Academy of Agricultural Sciences (SAAS), Shanghai, China. Of these, 195 accessions and their genome sequence have been reported in a previous study to uncover the genetic architecture for gummosis disease [17]. All trees were grafted on the wild rootstock ‘Mao Tao’, planted in 2016 and then managed under uniform conditions of irrigation, fertilization, and pest and disease control. Fruit samples were harvested at the commercial maturity stage determined by the changes of skin color as well as firmness in 2019. For each accession, flower type and pollen sterility were phenotyped and recorded by visual inspection at the flower blossom time in March. As for fruit flavor traits, a minimum of 20 homogeneous fruits, uniform in size and damage-free, were picked and immediately transported to the laboratory to evaluate fruit shape, fruit skin hairiness, flesh color, and soluble solids content. Soluble solids content was measured using a digital refractometer (ATAGO, PR-101α). The peel and mesocarp pulps of the samples were then separated, frozen immediately in liquid nitrogen and stored at −80°C for VOC evaluation [47].

### Phenotyping for volatile organic compounds

As published previously [58], five grams of frozen pulp powder were added to 20-ml vials containing 3 ml 200 mM ethylene diamine tetraacetic acid (EDTA) and 3 ml 20% CaCl_2_. Ten microliters of 2-octanol (0.8 g/l) was added as an internal standard and the sample was vortexed for 1 minute. The vials were incubated for 20 minutes in a solid-phase microextraction (SPME) autosampler coupled to an Agilent 7890A gas chromatograph and a 5977B mass spectrometer. Volatiles were collected using 65 μm PDMS–DVB autosampler StableFlex™ SPME fibers (Supelco Co., USA), then desorbed at the injector for 5 minutes at 240°C. The initial oven temperature was kept at 40°C for 1 minute, then raised first to 100°C at a rate of 3°C min^−1^ then to 220°C at a rate of 5°C min^−1^, held for 5 minutes, then raised to 245°C at a rate of 25°C min^−1^, and held for 1 minute. The injection, transfer line, ion source and quad temperatures were 250, 250, 230, and 150°C, respectively. The energy was 70 eV in electron impact mode. The mass spectrometry data were acquired under SIM mode with the scan range from 20–400 mZ^−1^. Volatile absolute mass concentration was calculated based on the standard curves created by the reference value of the internal standard and authentic compounds from Sigma chemicals (www.sigmaaldrich.cn). To adapt multiple-trait analysis and GWAS to numeric trait values, all quantity traits were normalized (mean 0, variance 1), so the quantity trait values could be compared together. The qualitative traits values were coded as 0, 1 for binomial distribution traits, and −1, 0, and 1 for trinomial distribution traits.

### Genotyping and genome-wide association study

Leaf collection, total genomic DNA isolation, construction of the genome re-sequence libraries, and SNP filtration were performed according to Li *et al*. [17]. The phenotypic and genotypic data obtained for each accession were subjected to GWAS using the mixed linear model (MLM) [59], FarmCPU [60], the multiple loci mixed linear model (MLMM), and BLINK [61] using GAPIT 3 in R [62]. We used principal components as covariates to evaluate familial relatedness and the kinship matrix to eliminate false-positive GWAS. Individual relationships were estimated using Zhang’s method in the GAPIT3 software [63]. The Bonferroni cutoff (0.01/the total number of markers) was used as the permutation cutoff threshold for adjustment of significant markers. Therefore, the cutoff threshold values should be 6.86e−8 (0.01/145456) in this study. For convenience of filtering data, we selected −log10(*P*) value >7 as threshold for adjustment of significant markers that were associated with targeted traits [64].

### Candidate gene prediction

Based on the linkage disequilibrium (LD) level in different subpopulations in our previous study on peach gummosis disease [17], candidate genes along the peach genome v2.0 from the Genome Database for Rosaceae (www.rosaceae.org) were identified by scanning the 100-kb flanking region on each side of the significant SNP. We used the RNA-seq data of the cultivars ‘Jin Chun’ (yellow-flesh peach) and ‘Hikawa Hakuho’ (white-flesh peach) during different developmental and ripening stages from our previous published studies (NCBI SRA database, PRJNA828349) for related gene identification.

### Genomic prediction

GP was estimated by the ridge regression best linear unbiased prediction (rrBLUP) method with the rrBLUP package in R version 4.0.5. Here, we estimated the proportion of genotype-explained variance in the total phenotype variance as narrow-sense heritability [65]. The variance component was estimated using the EMMA method in GAPIT [66, 67]. Other converting and transmitting codes were written by our research group with R (https://github.com/jiabowang/Multiple-Traits-GWAS).

Based on the GWAS results, the significant markers above the Bonferroni threshold were used to predict individual GEBVs with rrBLUP. Prior to GWAS, the whole population was randomly separated into five subpopulations. In each loop of cross-validation, four of the subpopulations were used in GWAS to detect significant markers that would be further used in training populations. The phenotype values of the fifth subpopulation, as the inference population, were used to calculate prediction accuracy. The Pearson’s correlation coefficient (*r*) between the GEBVs estimated from the reference set and phenotypic values in the inference set was defined as the accuracy. The random selection of individuals from the whole population was named as one-time cross-validation. The loop was the prediction in each of the five subpopulations. In this study, using 30 replicates, the average correlation between real phenotype and GEBVs was considered the final accuracy.

For the six agronomic traits (fruit flesh color, fruit shape, fruit skin hairiness, flower type and pollen sterility, and soluble solids content), the pseudo-QTNs (QTN, quantitative trait nucleotide) (selected by GWAS) were used as the random effect in the rrBLUP model.

For complex traits (*cis*-3-hexenyl acetate, linalool, β-ionone, γ-hexalactone, γ-octalactone, γ-decalactone, δ-decalactone, and γ-dodecalactone), the multiple QTNs (selected by GWAS in the relative traits) and the pseudo-QTNs (selected by GWAS in this trait) were used as the random effect in the rrBLUP model.

### Development, validation, and application of diagnostic DNA markers

Based on the GWAS result, two types of DNA markers were developed for MAS. The first, KASP markers, were designed for skin hairiness and fruit flesh color. Considering that the sample size of non-showy flower type and pollen sterility accessions was low, we did not design markers for these traits. Based on the identified SNPs, KASP primers were designed using PolyMarker and synthesized by Shanghai Sangon Biotech Co., Ltd (Supplementary Data Table S10). The PCR reaction was in 384-well PCR plates with 1.6 μl total mix volume including 20 ng/μl genomic DNA (0.8 μl), and 2 × KASP Master Mix (0.8 μl). The PCR program was as follows: 94°C for 15 minutes and 10 touchdown cycles of 94°C for 20 seconds and 61–55°C for 60 seconds (decreasing by 0.6°C per cycle). The second PCR amplification program included 26 cycles of 94°C for 20 seconds and 55°C for 60 seconds. The PCR reactions were performed in a Hydrocycler16 water instrument (LGC Genomics, Beverly, USA), and PCR fluorescence was detected by a PHERAstar (BMG LABTECH, Germany) microplate reader. We used KlusterCaller software (LGC Genomics, Beverly, USA) to analyze the data.

The second type of marker was an SSR marker designed for fruit linalool evaluation, with an AT motif repeat in the third intron of the GWAS-identified terpene synthesis gene (*PRUPE.4G030300*) (Supplementary Data Table S10). Both forward and reverse primers were synthesized, and the fluorescence label was added at the 5′ end of the forward primer. The polymorphism of the functional marker was amplified in 127 randomly selected accessions which were used in GWAS analysis. One-way ANOVA was used to compare the linalool content of different groups of genotypes. The PCR reaction system included: 1.0 μl 20 ng/μl DNA, 5.7 μl sterilized water, 2 μl 10 × PCR buffer (Mg^2+^), 0.20 μl dNTP (10 mmol/l), 0.50 μl of each forward and reverse primer (10 mmol/l), and 0.10 μl Taq polymerase. PCR conditions were set as in the previous method [1] except that the annealing temperature was modified to 53°C. The PCR fragments were analyzed using an ABI Prism 3730 capillary sequencer, and fragment sizes were called by Gene Mapper^®^ (Applied Biosystems, by Life Technologies).

## Supplementary Material

Web_Material_uhad117

## Data Availability

The datasets presented in this study are available in the tables, figures and supplementary tables. The original genome resequencing data and the RNA-seq data are publicly available in National Center for Biotechnology Information (NCBI) BioProject database under accession number PRJNA746706 and PRJNA828349.
