# Supplementary figures and images for: Multiple-statistical genome-wide association analysis and genomic prediction of fruit aroma and agronomic traits in peaches
Source: Hortic Res. 2023 May 31;10(7):uhad117. doi: 10.1093/hr/uhad117 (PMC10419450; doi:10.1093/hr/uhad117)

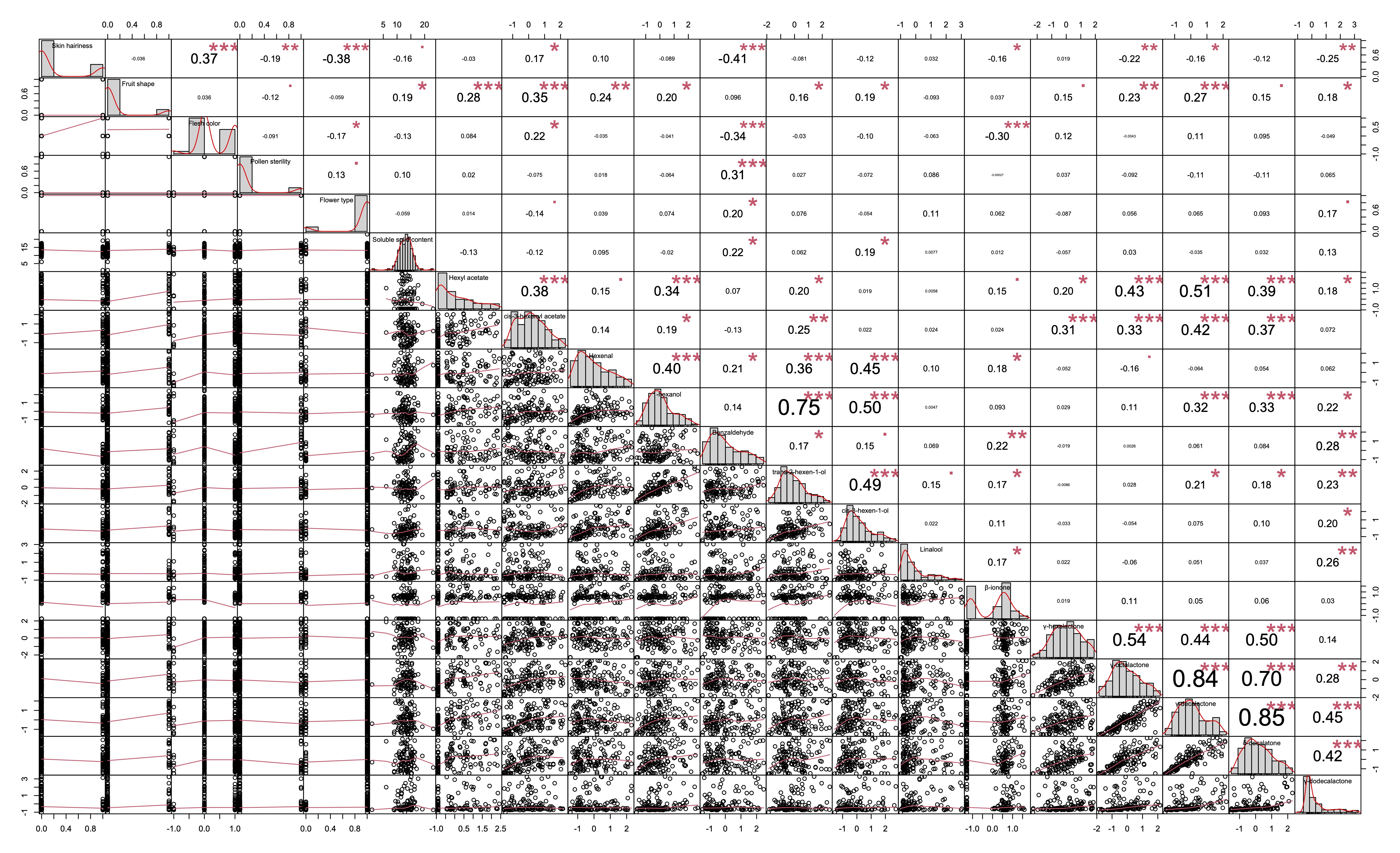

Supplement: Web_Material_uhad117 [file web_material_uhad117.zip › Figure_S1.jpg]

## Slide 1
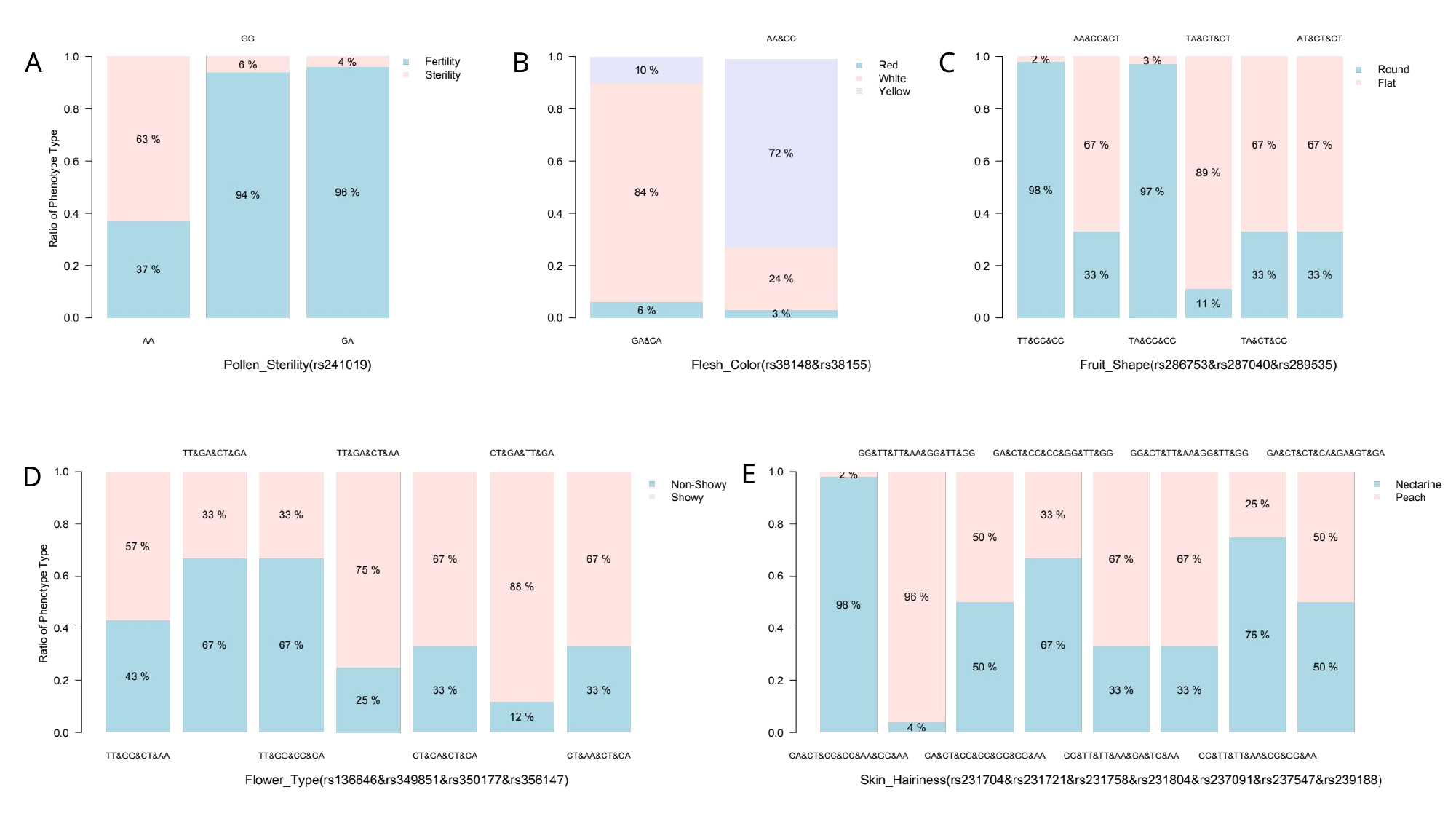

A
 B
 C
 E
 D

Supplement: Web_Material_uhad117 [file web_material_uhad117.zip › Figure_S2.pptx]

## Slide 1
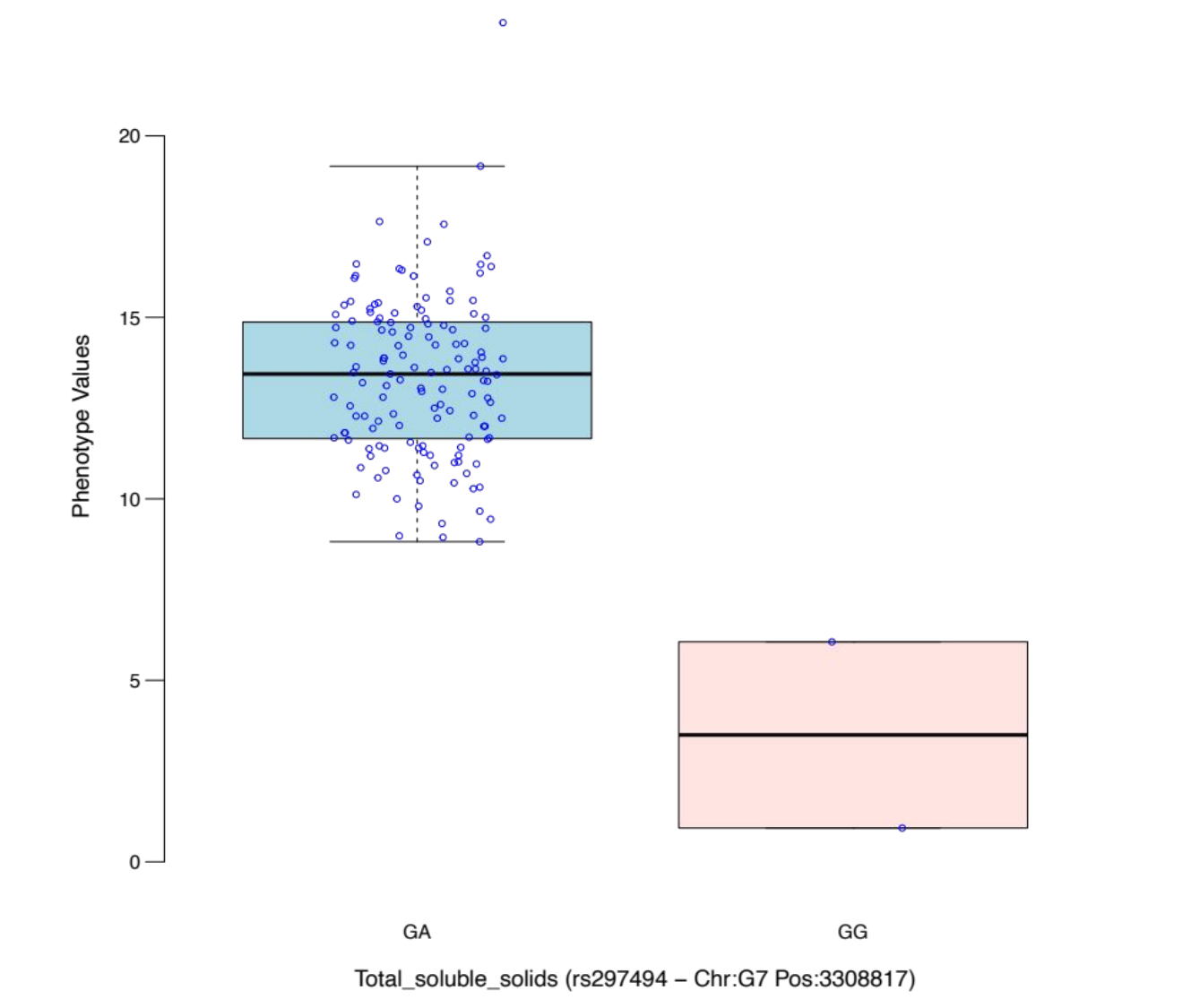

Supplement: Web_Material_uhad117 [file web_material_uhad117.zip › Figure_S3.pptx]

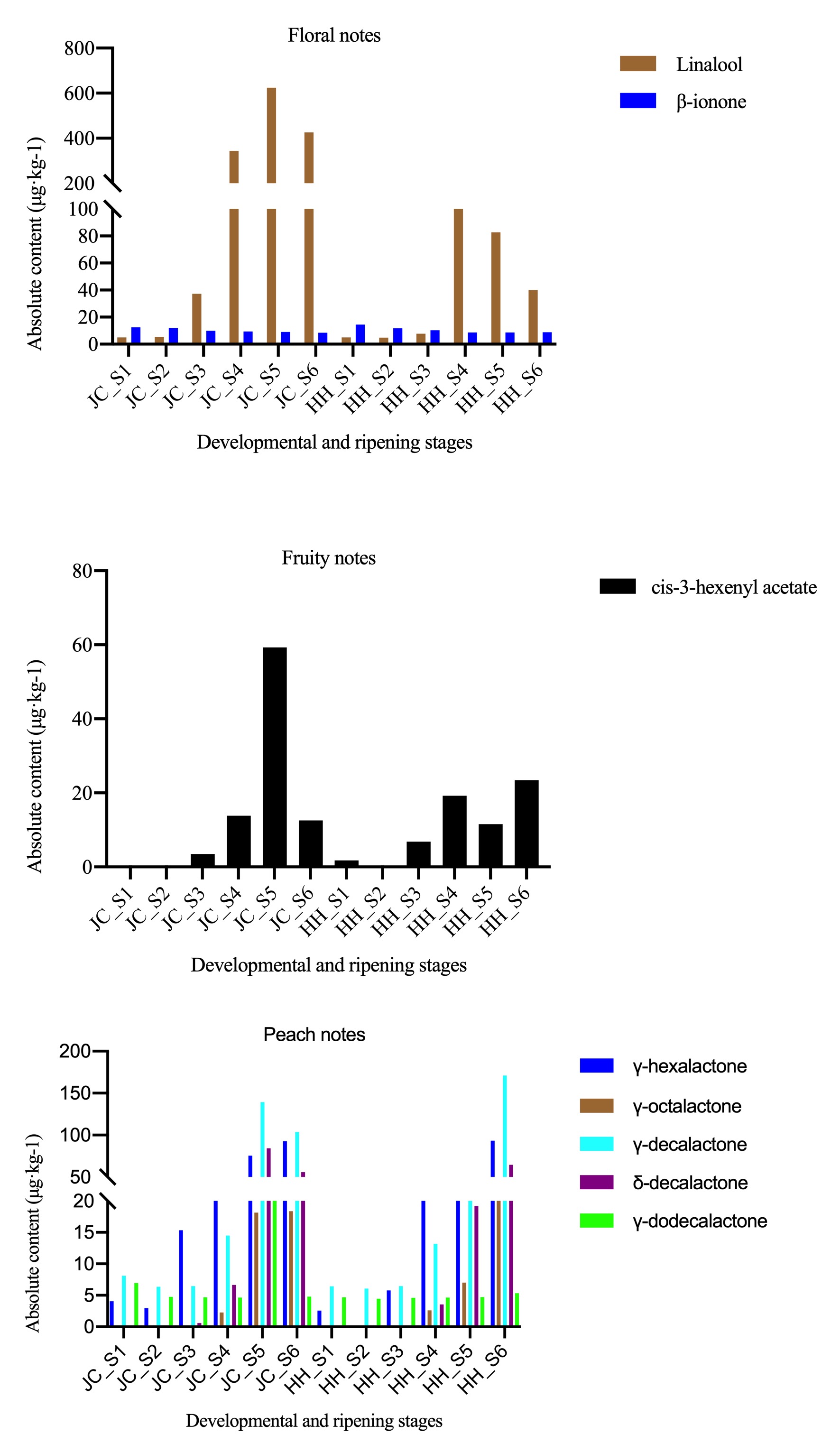

Supplement: Web_Material_uhad117 [file web_material_uhad117.zip › Figure_S4.jpg]

## Slide 1
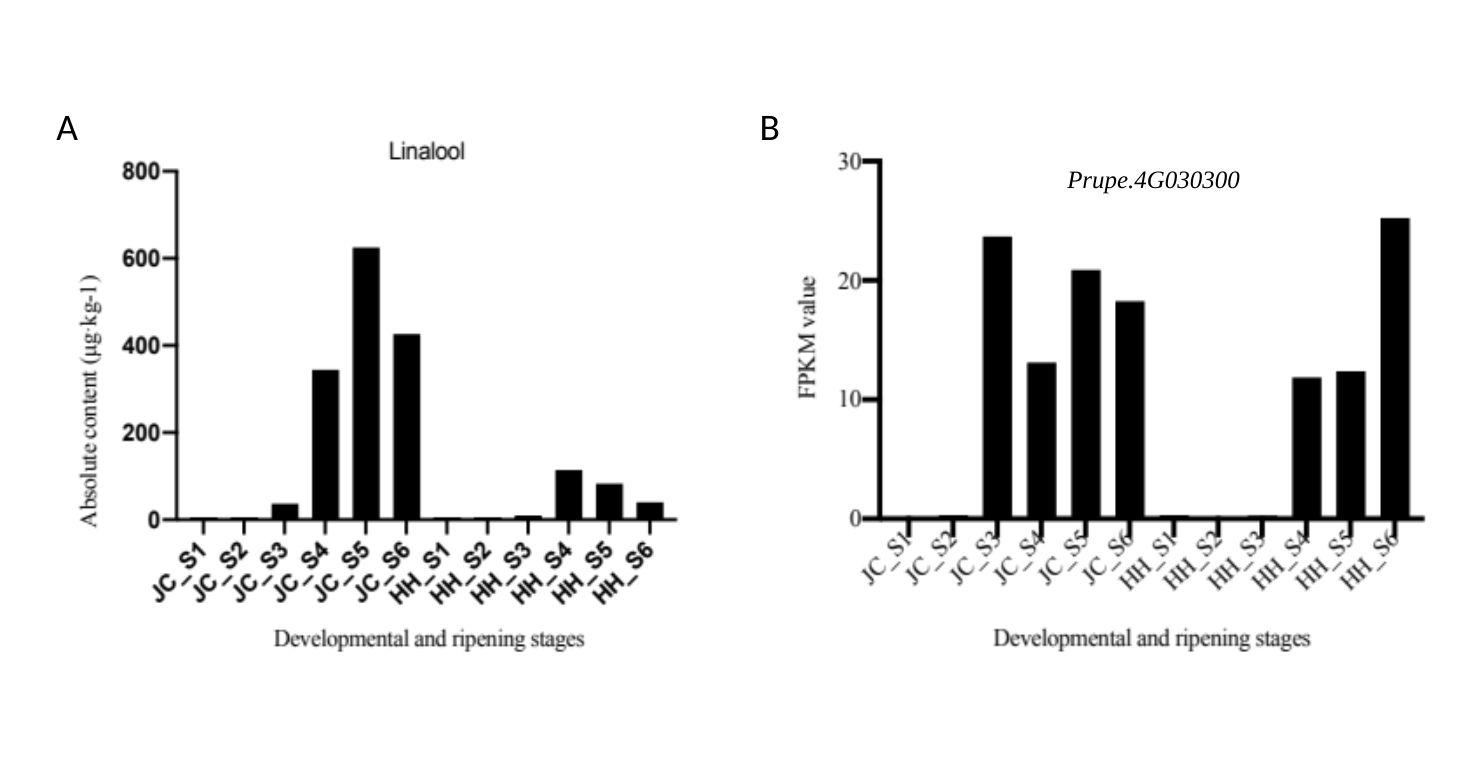

A
B
Prupe.4G030300

Supplement: Web_Material_uhad117 [file web_material_uhad117.zip › Figure_S5.pptx]

## Slide 1
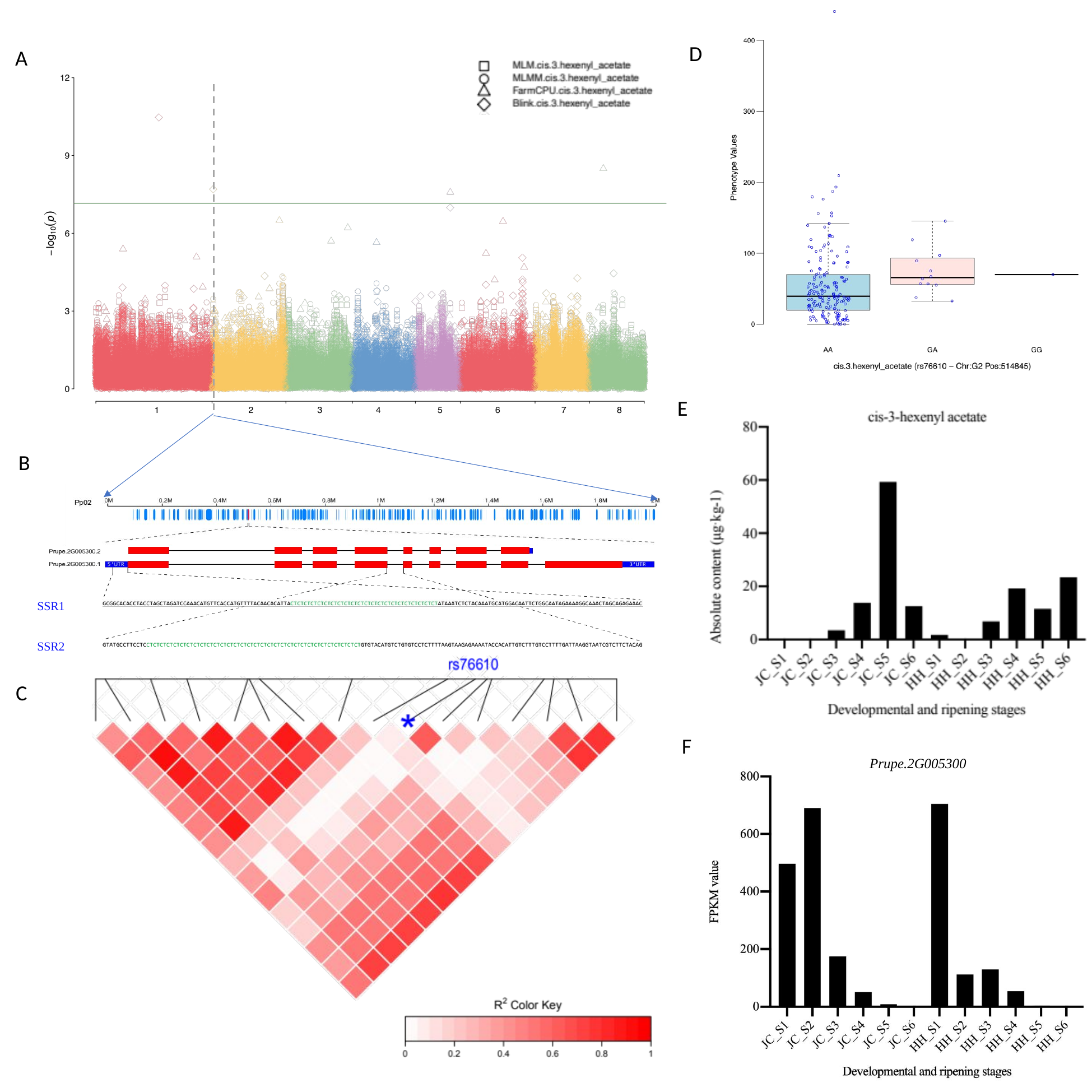

D
A
E
SSR1
SSR2
B
C
F
Prupe.2G005300

Supplement: Web_Material_uhad117 [file web_material_uhad117.zip › Figure_S6.pptx]

## Slide 1
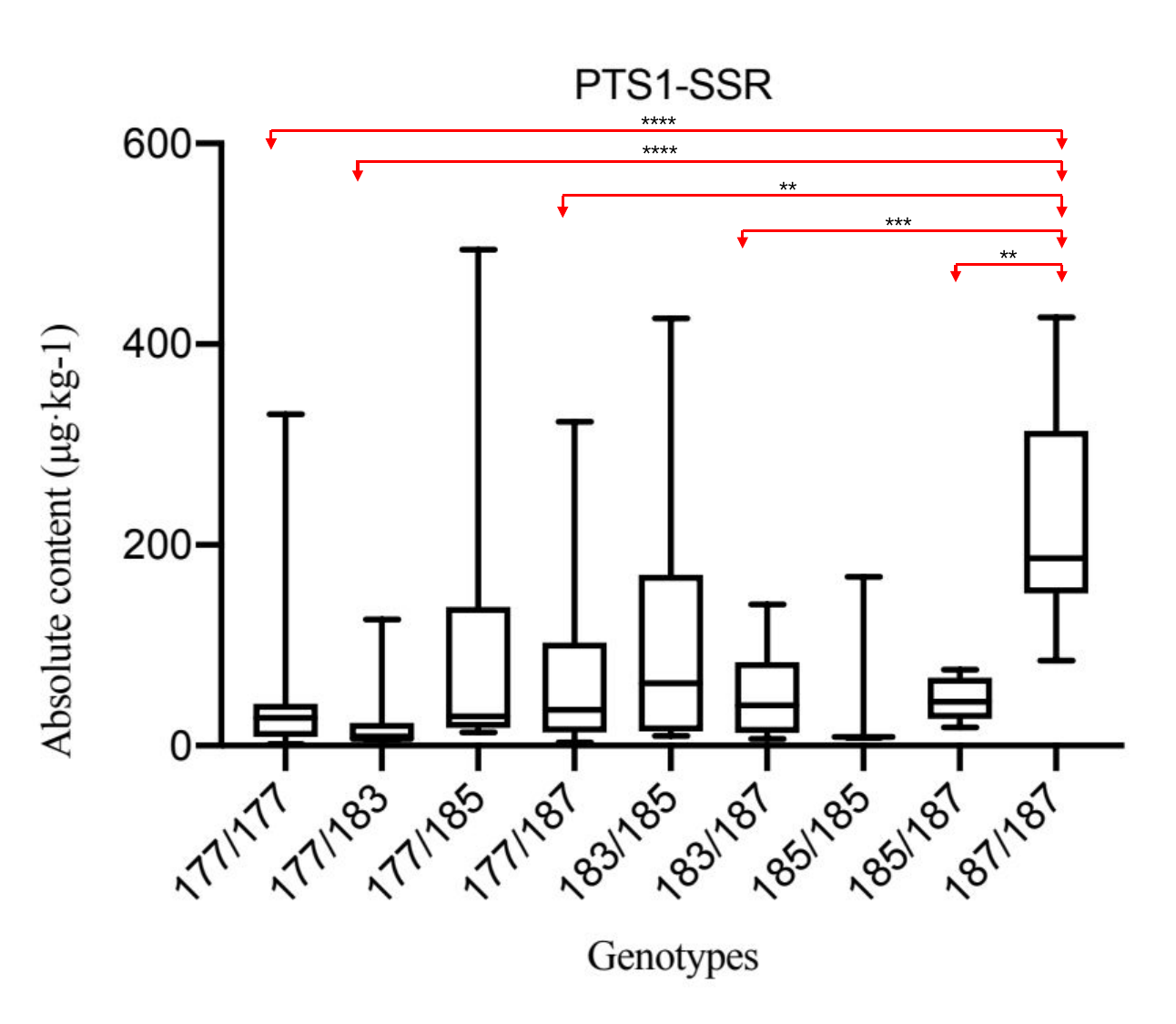

| \*\*\*\* |
| --- |
| \*\*\*\* |
| --- |
| \*\* |
| --- |
| \*\*\* |
| --- |
| \*\* |
| --- |

Supplement: Web_Material_uhad117 [file web_material_uhad117.zip › Figure_S7.pptx]
